# Basophil Activation Test Using Recombinant Allergens: Highly Specific Diagnostic Method Complementing Routine Tests in Wasp Venom Allergy

**DOI:** 10.1371/journal.pone.0108619

**Published:** 2014-10-17

**Authors:** Lukas Balzer, Davide Pennino, Simon Blank, Henning Seismann, Ulf Darsow, Mathias Schnedler, Mareike McIntyre, Markus W. Ollert, Stephen R. Durham, Edzard Spillner, Johannes Ring, Liliana Cifuentes

**Affiliations:** 1 Department of Dermatology and Allergy, Biederstein, Technische Universität München, Munich, Germany; 2 ZAUM – Center of Allergy and Environment (ZAUM), Technische Universität and Helmholtz Center Munich, Munich, Germany; 3 Institute of Biochemistry and Molecular Biology, University of Hamburg, Hamburg, Germany; 4 Molecular Immunology, Department of Allergy and Clinical Immunology, National Heart and Lung Institute, Imperial College, London, United Kingdom; 5 Department of Engineering, Immunological Engineering, Aarhus University, Aarhus, Denmark; 6 Christine Kuhne-center for Allergy Research and Education (CK-CARE), Davos, Switzerland; King's College London, United Kingdom

## Abstract

**Background:**

Skin testing can expose allergic subjects to potential systemic reactions, sensitization against unrelated proteins, and increased risk of future sting reactions. Therefore the continuous improvement of *in vitro* diagnostic methods is desirable. Recombinant allergens have been shown to improve the sensitivity of specific IgE (sIgE) detection *in vitro* whilst no data is available regarding their application and reliability in basophil activation test (BAT). Here we aimed to compare the specificity and sensitivity of recombinant allergens Ves v 1, Ves v 2, Ves v 3 and Ves v 5 in both specific IgE (sIgE) detection in *vitro* and basophil activation test.

**Methods:**

sIgE detection by ELISA or ImmunoCAP and BAT towards the panel of recombinant allergens Ves v 1, Ves v 2, Ves v 3 and Ves v 5 were performed in 43 wasp venom allergic patients with a history of anaphylactic reaction and sIgE seropositivity, as well as 17 controls defined as subjects with a history of repetitive wasp stings but absence of any allergic symptom.

**Results:**

The BAT performed with the recombinant allergens Ves v 1, Ves v 2, Ves v 3 and Ves v 5 markedly improved the specificity of diagnosis in wasp venom allergic subjects when compared to the respective sIgE detection in serum.

**Conclusions:**

BAT performed with the recombinant allergens Ves v 5, Ves v 3 and Ves v 1 provides an emerging highly specific *in vitro* method for the detection of wasp venom allergy, compared to the sIgE detection. Recombinant allergens applied to BAT represent a step forward in developing reliable *in vitro* tests for specific diagnosis of allergy.

## Introduction

Up to 3% of the general population suffer from potentially life-threatening systemic reactions after wasp stings [1]. The diagnostic work-up and the indication for treatment by venom specific immunotherapy (SIT) is well established by the combination of allergy history and the detection of specific IgE (sIgE) to the suspected venom measured by skin testing and/or in *vitro* detection in serum [2], [3]. However, skin testing exposes allergic subjects to systemic reactions, sensitization against unrelated proteins, and increased risk of future sting reactions [4]–[8]. Moreover, skin conditions such as dermographism or severe skin diseases do not allow skin testing. Therefore, the continuous development of more specific and sensitive *in vitro* tests complementing the current *in vitro* sIgE detection is desirable. Recently, the *in vitro* diagnostic gap of serological sIgE detection [9], [10] has been improved by analysis of sIgE against wasp venom recombinant allergens [11].

Allergenic components from wasp venom have been characterized and expressed in recombinant forms. The wasp venom allergens that are thought to be primarily responsible for IgE-mediated allergic reactions include phospholipase A_1_ (Ves v 1) [12], hyaluronidase (Ves v 2) [13], [14], antigen 3 (Ves v 3) [15] and antigen 5 (Ves v 5) [16]. Among multiple allergens present in the hymenoptera venoms, the wasp venom antigen 5 (Ves v 5) has been shown to be present in high concentration in native extracts and to improve the serological *in vitro* diagnosis of wasp venom allergic patients [16]–[18].

Although sIgE toward underrepresented recombinant allergens identify allergic patients undetected with standard in vitro tests [11], [19], there is only little evidence for a risk of generating false positive results.

To further improve current diagnostic approaches, in *vitro* basophil activation (BAT) to wasp venom has been suggested to be a sensitive test complementing the diagnosis of insect venom allergy [20], [21]. However, possible advantages of the application of underrepresented recombinant allergens in BAT have not been investigated.

Therefore, the aim of this study was to evaluate the specificity and sensitivity of a panel of recombinant allergens Ves v 1, Ves v 2, Ves v 3 and Ves v 5 in both serum sIgE detection and basophil activation test.

## Results

### Recombinant allergens activate basophils in allergic patients

**Figure 1:**
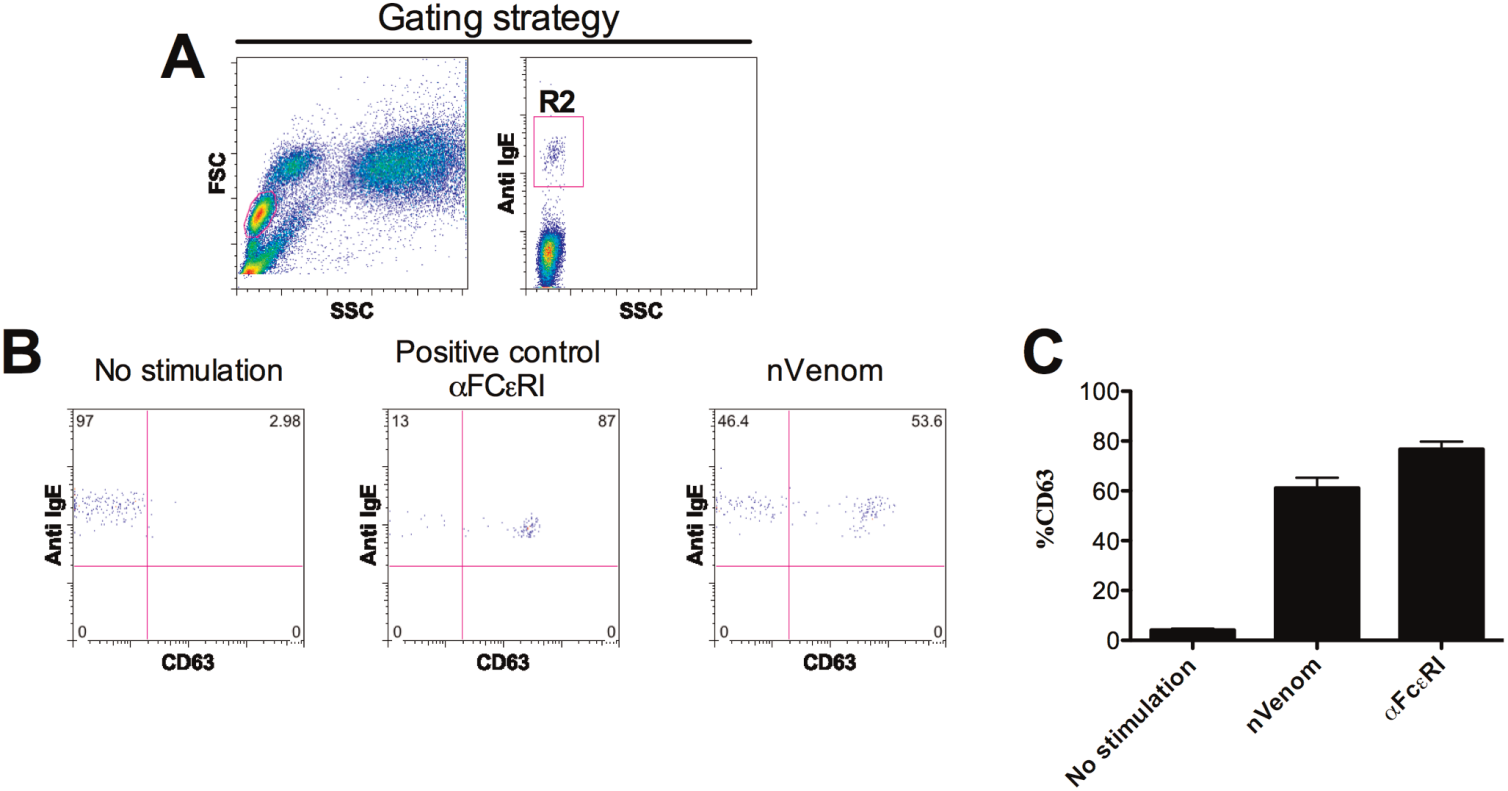
Wasp venom extracts activate basophils in the whole blood of allergic patients. (**A**) Identification of basophils according to dimension and IgE levels. (**B**) Representative two-color flow cytometric staining dot plots of IgE and CD63 on basophils unstimulated, stimulated with anti-FCεRI and nVenom. (**C**) CD63 expression on basophils in absence of stimulation, stimulated with anti-FCεRI and nVenom. Scale bars indicate mean ± SD.

**Figure 2:**
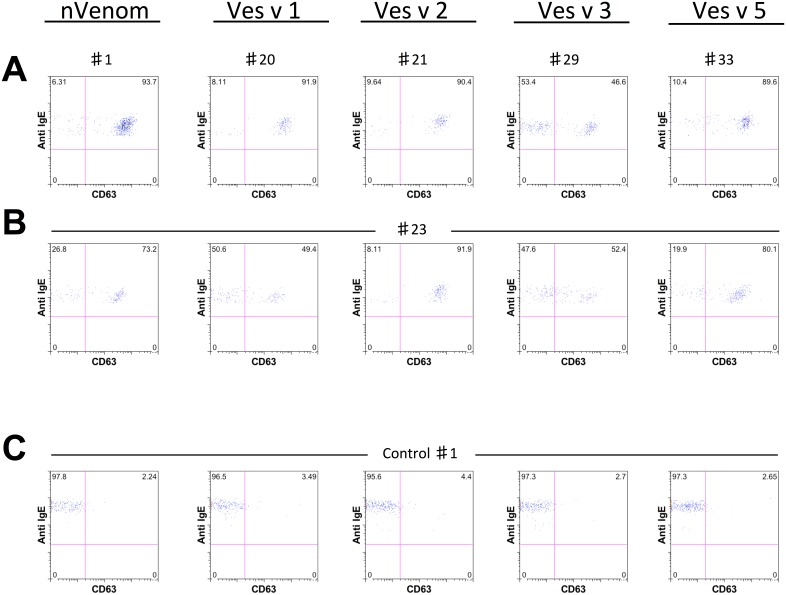
Recombinant allergens strongly activate basophils in allergic subjects. Representative two-color flow cytometric staining dot plots of IgE and CD63 on basophils of (**A**) five representative allergic subjects responsive to each recombinant allergen, (**B**) a representative multi-sensitized patient responsive to all the recombinant allergens (**C**) degree of basophil activation in a representative subject of the control group.

To explore whether wasp venom extracts (nVenom) and the recombinant allergens Ves v 1, Ves v 2, Ves v 3 and Ves v 5 activate basophils; we performed the basophil activation test in wasp venom allergic and non-allergic subjects (Table 1). Basophils were identified according to their dimension and IgE positivity (Figure 1a). Activation of basophils was measured as percentage of basophils expressing CD63. The stimulation of basophils with the positive control: monoclonal antibody recognizing the high affinity IgE binding receptor (IgE-FCεRI) as well as natural venom, indicated that basophils were in good conditions during the experiments and that natural venom activated basophils in allergic subjects (Figure 1b and 1c). Furthermore, we evaluated the degree of basophil activation towards recombinant allergens in wasp venom allergic subjects. rVes v 1, rVes v 2, rVes v 3 and rVes v 5 strongly activated basophils in responsive subjects (Figure 2a and 2b). The degree of basophil activation was largely different between allergic and control subjects. Whilst in some allergic subjects recombinant allergens induced over 90% of CD63 expression (Figure 2a), in non-allergic subjects the degree of CD63 expression on basophil was lower than 5% (Figure 2c). In few cases, the expression of CD63 on basophils in the control group was higher than 5% but always less than 15.7%. These data indicate that recombinant allergens fully activate basophils and that allergen induced CD63 expression clearly distinguishes between allergic and non-allergic subjects.

**Table 1:** Patient characteristics.

	Age	Sex(M/F)	Experience of wasp sting	Total IgE KU/L(Mean±SD)	Specific IgEKU/L(Mean±SD)
Wasp venom allergic patients	48.8±18	20/23	43/43	396.2±901	14.4±22.3
Control group	29.00±7.303	9/8	17/17	140.6±220	0.2147±0.5

### BAT improves specificity of the panel of recombinant allergens Ves v 1, Ves v 2, Ves v 3 and Ves v 5

To compare specificity and sensitivity of recombinant allergens in both sIgE detection and BAT, we analyzed receiver operator curves (ROC) by comparing allergic and non allergic patients (Table 1) of both tests performed with recombinant allergens Ves v 1, Ves v 2, Ves v 3 and Ves v 5.

**Figure 3:**
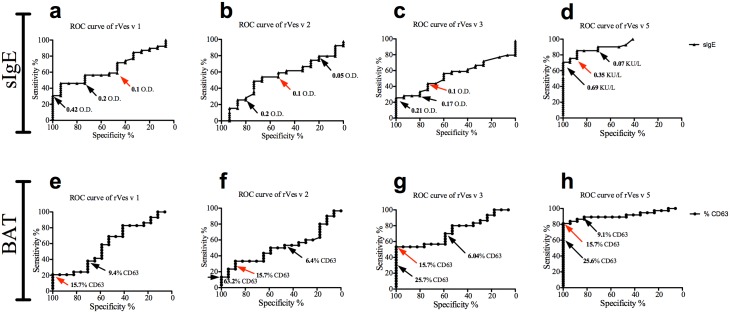
Recombinant allergens improve the *in vitro* test specificity for the identification of wasp venom allergic patients. ROC curves of specific IgE measurement (sIgE) of recombinant Ves v 1, Ves v 2 and Ves v 3 by ELISA (O.D.) (**A–C**) and recombinant Ves v 5 by ImmunoCAP (KU/L) (**D**). ROC curves of CD63% trade-off of basophil stimulated with recombinant Ves v 1, Ves v 2, Ves v 3 and Ves v 5 (**E–H**).

ROC curves of BAT indicate the sensitivity and specificity at different cut-off of basophil activation measured as CD63 expression on basophils. ROC curves of allergen-specific-IgE indicate the specificity and sensitivity of specific-IgE measurement at different O.D. or KU/L levels. Measurement of sIgE to recombinant Ves v 5 showed sensitivity of 78.05% and specificity of 88.24% at the cut-off of 0.35 KU/L. Since 11.76% of the patients having sIgE to rVes v 5 were false positive and 21.95% false negative (Figure 3d), we further analyzed the sIgE against every recombinant allergen. Measurement of sIgE to rVes v 1, rVes v 2 and rVes v 3 (Fig. 3a, b and c) showed a weak trade-off of specificity *vs.* sensitivity compared to rVes v 5.

BAT towards the recombinant allergens Ves v 3 and Ves v 5 strongly improved the trade-off of specificity *vs* sensitivity when compared to the respective sIgE analysis. When 15.7% of the basophils expressed CD63 after antigen stimulation, rVes v 5 showed 100% specificity and 81.57% sensitivity (Figure 3h) while rVes v 3 100% specificity and 53.33% sensitivity (Figure 3g). At the cut-off of 15.7% rVes v 2 (Figure 3f) and rVes v 1 (Figure 3e) improved the specificity but diminished the sensitivity compared with the respective sIgE detection. These data suggest that recombinant allergens applied to BAT represent a very specific tool to improve *in vitro* diagnosis of wasp venom allergic patients.

### Recombinant allergens differentially detect allergic patients in basophil activation test

Venom allergic patients were analyzed for sIgE and BAT to natural venom and the panel of recombinant allergens.

The grade of concordance between sIgE and BAT was high in the case of commercial venom extract and rVes v 5 (Table 2). Patients were positive for both sIgE and BAT in 67.8% (38/56) of cases for conventional venom extract, 79.2% (42/53) for rVes v 5, 58.5% (24/41) for rVes v 3, 56.1% (23/41) for rVes v 2 and 60% (24/40) for rVes v 1. Interestingly, patients negative for natural venom in BAT were often positive for rVes v 5 and/or rVes v 1, rVes v 2 and rVes v 3 (Table 3). Conversely, some patients negative to rVes v 5 in BAT were positive to natural venom and/or other allergens (Table 3). Most of the patients showed positivity to more than one allergen in BAT as well as sIgE detection. Being the BAT a very specific test, this multi-positivity suggests that some patients such as #23 and #41 are multi-sensitized while others reacted only to one allergen such as #18 and #19 might be mono-sensitized.

**Table 2:** Grade of concordance of specific IgE (sIgE) in serum and Basophil activation test (BAT).

Grade of concordance sIgE *vs* BAT				
	Allergic patients		Total subjects	
	Ratio	spearman test	Ratio	spearman test
nVenom	65.8% (27/41)	0.194	67.8% (38/56)	0.48***
rVes v 5	75% (27/36)	0.158	79.2% (42/53)	0.6057***
rVes v 3	48% (12/25)	−0.2785	58.5% (24/41)	−0.2036
rVes v 2	56% (14/25)	−0.2543	56.1% (23/41)	−0.2735
rVes v 1	68% (17/25)	−0.1617	60%% (24/40)	−0.0645

Statistically significant differences were defined as *P<0.05, **P<0.01 and ***P<0.001.

**Table 3:** Recombinant allergens differentially recognize venom allergic patients.

Allergic Patients											
		Specific IgE					BAT				
Patient	Skintest	nVenom≥0.35 KU/L	V1≥0.1 O.D	V2≥0.1O.D.	V3≥0.1O.D.	V5≥0.35 KU/L	nVenom≥15.7%	V1≥15.7%	V2≥15.7%	V3≥15.7%	V5≥15.7%
1	+	+	+	−	−	+	+	N.D.	N.D.	N.D.	+
2	+	+	+	−	−	+	−	N.D.	N.D.	N.D.	−
3	+	+	+	−	−	+	+	N.D.	N.D.	N.D.	+
4	+	+	+	+	+	−	N.D.	N.D.	N.D.	N.D.	N.D.
5	+	+	+	−	+	+	−	N.D.	N.D.	N.D.	+
6	+	+	+	+	+	+	+	N.D.	N.D.	N.D.	+
7	+	+	+	−	−	+	+	N.D.	N.D.	N.D.	−
8	+	+	+	+	+	+	+	N.D.	N.D.	N.D.	+
9	+	+	+	−	+	+	+	N.D.	N.D.	N.D.	+
10	+	+	+	+	+	+	+	N.D.	N.D.	N.D.	+
11	+	+	−	−	−	−	+	N.D.	N.D.	N.D.	N.D.
12	+	+	−	−	−	−	+	−	−	−	+
13	+	+	−	+	+	−	−	−	−	−	−
14	+	+	−	−	−	+	+	−	−	+	+
15	+	+	N.D.	N.D.	N.D.	+	+	−	−	−	+
16	+	+	−	−	+	+	+	−	−	+	+
17	+	+	N.D.	N.D.	N.D.	+	+	−	+	+	+
18	+	+	−	−	−	+	+	−	−	−	+
19	+	+	−	−	−	+	+	−	−	−	+
20	+	+	−	−	−	+	+	−	+	+	+
21	+	+	−	−	−	+	+	−	+	+	+
22	+	+	+	−	−	+	N.D.	N.D.	N.D.	+	N.D.
23	+	+	+	+	+	+	+	+	+	+	+
24	+	+	−	−	−	+	−	−	−	−	+
25	+	+	+	+	+	+	−	−	−	−	+
26	+	+	−	+	+	N.D.	−	−	−	+	+
27	+	+	−	−	+	+	−	−	−	+	+
28	+	+	+	−	−	N.D.	−	−	−	+	+
29	−	+	+	+	+	−	−	+	+	+	+
30	+	+	+	−	−	+	+	+	+	+	+
31	+	+	+	+	−	+	+	+	+	−	+
32	+	+	+	+	+	+	−	−	−	−	−
33	+	+	N.D.	N.D.	N.D.	+	+	−	−	−	+
34	+	+	+	−	−	+	+	−	−	−	+
35	+	+	+	+	+	−	+	−	−	−	+
36	+	+	+	−	−	+	+	+	−	+	−
37	+	−	N.D.	N.D.	N.D.	+	+	+	+	+	+
38	+	+	+	+	+	+	+	N.D.	N.D.	N.D.	N.D.
39	−	+	−	+	−	−	−	−	−	−	−
40	+	+	N.D.	N.D.	N.D.	+	−	−	−	−	−
41	+	+	+	+	−	+	+	−	+	+	+
42	+	+	−	−	−	−	+	+	+	+	+
43	+	+	+	+	+	−	−	−	−	N.D.	N.D.
**Control Group**											
1	N.D.	−	−	−	−	−	−	−	−	−	−
2	N.D.	N.D.	N.D.	−	−	−	−	−	−	−	−
3	N.D.	−	+	+	+	−	−	−	+	−	−
4	N.D.	−	−	−	−	−	−	−	−	−	−
5	N.D.	−	−	−	−	−	−	−	−	−	−
6	N.D.	−	−	−	−	−	−	−	+	−	−
7	N.D.	−	N.D.	N.D.	N.D.	+	+	−	−	−	−
8	N.D.	−	+	+	−	−	−	−	−	−	−
9	N.D.	−	−	−	−	−	−	−	−	−	−
10	N.D.	N.D.	+	+	−	−	−	−	−	−	−
11	N.D.	−	−	−	−	−	−	−	−	−	−
12	N.D.	N.D.	−	−	−	−	−	−	−	−	−
13	N.D.	−	+	+	−	−	−	−	−	−	−
14	N.D.	−	+	+	+	−	−	−	−	−	−
15	N.D.	+	+	+	+	−	−	−	−	−	−
16	N.D.	+	+	+	+	−	−	−	−	−	−
17	N.D.	+	+	−	−	+	−	−	−	−	−

*Table legend*: nVenom =  natural venom, V1 =  rVes v 1 V2 =  rVes v 2 V3 =  rVes v 3, V5 =  rVes v 5; “+” =  positive patient; “–“ =  negative patient; N.D. =  not done: sIgE =  specific IgE; BAT =  Basophil activation test. Cut-offs: 0.35 KU/L ImmunoCaps, 0.1 O.D. ELISAs, 15.7% CD63 expression in BAT. Empty spaces indicate untested samples.

### Component-resolved-analysis tends to increase sensitivity of basophil activation test: proof of principle

**Figure 4:**
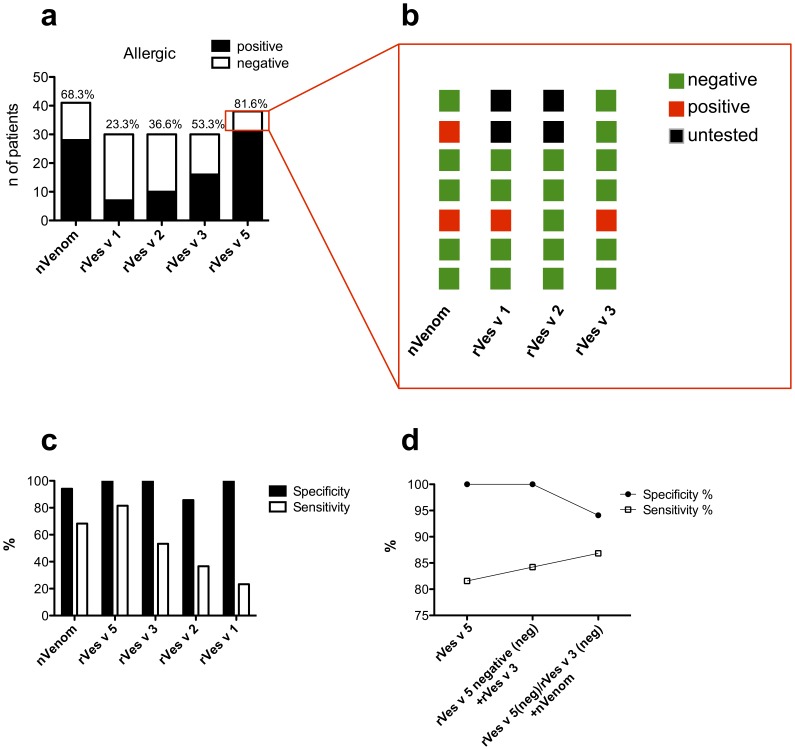
Analysis of patients negative to rVes v 5 with rVes v 3, rVes v 1 and natural venom, improves the trade-off between sensitivity and specificity of the Basophil activation test. (**A**) Percentage of patients positive to basophil activation test performed with natural venom and recombinant allergens. (**B**) Analysis of patients negative to recombinant Ves v 5 with natural venom and other recombinant allergens. (**C**) Specificity and sensitivity of natural venom, recombinant Ves v 5 and recombinant Ves v 3 at cut off 15.7% CD63 expression. (**D**) Specificity and sensitivity of multistep analysis of BAT considering rVes v 5 alone, rVes v 5 plus rVes v 3, and rVes v 5 plus rVes v 3 plus natural venom.

BAT with rVes v 5 at 15.7% CD63 expression provide a specificity of 100% and sensitivity of 81%. Since some wasp venom allergic patients resulted negative to BAT performed with rVes v 5 but positive to other recombinant allergens (Table 3) we evaluated whether a further improvement of the trade-off between sensitivity and specificity of the BAT could be achieved by analyzing rVes v 5 negative patients with natural venom and others recombinant allergens. ROC curve analysis indicated that the CD63 expression of 15.7% is the best common cut-off among recombinant allergens. While BAT performed with natural venom showed a specificity of 94.1% and sensitivity of 68.3%, BAT performed with rVes v 5 followed by rVes v 3 were the most sensitive and specific among the recombinant allergens tested (Figures 4a and 4c).

Patients negative to rVes v 5, resulted positive to rVes v 3 (1/7) or conventional venom extract (2/7) (Figures 4a and 4b). The analysis of rVes v 5 negative patients with rVes v 3 maintained the specificity of 100% and increased the sensitivity from 80% up to 85% (Figure 4d). Patients negative for both rVes v 5 and rVes v 3 were further analyzed with conventional venom extract. This analysis increased the sensitivity of the allergological diagnosis up to 87% but slightly decreased the specificity from 100% to 94% (Figure 4d).

Together, these data indicate that the combination of recombinant allergens and natural venom tend to achieve a better trade-off between test specificity and sensitivity.

## Discussion

In the present study we validated the usefulness of the panel of recombinant allergens Ves v 1, Ves v 2, Ves v 3 and Ves v 5 in the identification of wasp venom allergic patients. Basophil activation test performed with recombinant allergens is a highly specific tool to identify wasp venom allergic patients. Interestingly, recombinant allergens are more specific and sometimes less sensitive when tested in BAT compared to the respective sIgE detection. However, the sensitivity of BAT tends to improve by the component-resolved testing of a panel of recombinant allergens.

The skin test is the gold standard method for allergy diagnosis. However, it exposes allergic subjects to potential systemic reactions, sensitization against unrelated proteins, and increased risk of future sting reactions [4]–[7]. On the other hand, patients classified as false negative after standard tests do not receive immunotherapy, and thus are at risk of systemic reactions in case of wasp sting.

Consequently, studies attempting to improve the quality of *in vitro* wasp venom allergic diagnosis have been increasingly published. Among these studies, two new approaches have been developed: the measurement of sIgE towards recombinant allergens [11], [19]; and the basophil activation test with the natural venom [20].

Specific IgE towards recombinant allergens detect the majority of wasp venom allergic patients resulted negative to sIgE against natural venom, improving detection of wasp venom allergic patients [11], [16]. However, the risk of generating false positive and negative results by the application of recombinant allergens has not been evaluated. We found that sIgE detection of rVes v 5 allows only 11.76% of false positive patients whilst the specificity of the other recombinant allergens was very limited.

Interestingly, recombinant allergens were much more specific when tested in basophil activation test compared to the respective sIgE detection. This finding might reflect the discrepancy between the sensitization state of patients and the allergic state, where sIgE should induce cellular activation. This result is in line with previous reports suggesting that sIgE may be not functional and detection of the functional IgE may improve the discrimination between allergic and atopic state of the patients. Presence of serum sIgE could be associated with the atopic status rather than the allergic status and IgE-mediated anaphylaxis. Specific antibodies are frequently detected in individuals with high total IgE, but appear to be often irrelevant in clinical terms and as many studies have indicated, atopy is not a risk factor for the development of hymenoptera venom allergy [22]. Therefore, the ability of sIgE to mediate cellular responses might differentiate between atopic and allergic status of subjects. A second possibility is a technical limitation possibly due to the solid phase of allergens in the immunoCAP and ELISA. Indeed, in this state the binding of different sIgE to distinct epitopes of the allergens might be inhibited. In this case BAT may represent a more physiological readout facilitating the binding between the cognate allergen and the sIgE.

Of note, use of recombinant Ves v 5 and Ves v 3 strongly increases both sensitivity and specificity of BAT compared to respective sIgE detection. Although rVes v 5 is highly expressed in conventional venom extract, some patients result positive to sIgE against rVes v 5 and negative to wasp venom extract. This paradoxical evidence leads to the hypothesis that different commercial extracts may differentially express Ves v 5. Extracts from different vespula species are mixed and thereby might induce very different BAT activation depending on the manufacturer and specific lot used [23]. Therefore, recombinant allergens might be applied in future for the harmonization of the BAT performed in different laboratories.

Of note, we found patients positive either to several recombinant allergens or single one. This finding could reflect the sensitization state of those patients and clinically translate into immunotherapies against a single allergen rather than the whole natural venom. In fact, a mono-sensitized patient could theoretically develop sensitization to other allergens determining the failure of the immunotherapy performed with the natural venom extract. Therefore, the identification of the mono-sensitized allergic patients might in future develop into a stratified and personalized immunotherapy.

In our cohort only two patients were negative to skin test, but positive to *in vitro* sIgE detection against natural venom complicating the diagnosis of wasp venom allergy. The BAT performed with recombinant allergens finds its usefulness in such cases. In fact, whilst our control group demonstrated that *in vitro* sIgE detection against natural venom and recombinant allergens generate false positive results; the BAT is more specific. Indeed, patient #29 negative to skin test is positive to BAT with rVes v 5. Being BAT a very specific assay, patient #29 can be included among patients undergoing immunotherapy.

However, the BAT shows its limits with patient #39. This patient is negative in skin test, positive to standard sIgE detection and negative to BAT performed with all recombinant allergens. The sensitivity of BAT performed with all the recombinant allergens did not ensure the detection of the totality of allergic subjects. It is possible that this patient is sensitized to an underrepresented allergen that has not been tested in this study. The generation and validation of further recombinant allergens increasing the sensitivity of BAT may lead to a further improvement of this assay.

Further studies aiming at investigating the patients negative to standard tests should be performed.

The panel of recombinant allergens complements the current diagnosis of wasp venom allergy by identifying multi-sensitized patients and improving the specificity of the *in vitro* test. Further generation of recombinant allergens may be a further step to improve the sensitivity of BAT and the detection of patients mono-sensitized to underrepresented allergens.

In conclusion, basophil activation test performed with recombinant allergens improves the specificity of *in vitro* diagnosis and may represent a step forward in developing a reliable *in vitro* test for the diagnosis of allergic patients.

## Materials and Methods

### Ethic statement

All clinical investigation has been conducted in accordance with the Helsinki Declaration. Blood from venom allergic patients with YJV-specific IgE and/or positive skin test results were collected during clinical practice. All donors had given their informed written consent and all experiments were approved by the local ethics committee of the faculty of medicine of the Technische Universität München, Munich, Germany (Technische Universität München – Fakultät für Medizin - Ethikkommission).

### Characterization of venom-allergic patients

Forty-three patients with clear history of systemic anaphylactic reactions, positive skin test and/or sIgE have been included in the study (Table 1). Severity of clinical symptomatology was evaluated according to a previously published score system [24]. Skin test, total IgE and specific IgE measurement were performed in every patient. IgE reactivity and effector cell responses were evaluated with natural venom (Buhlmann) and insect cell expressed allergens, including non-glycosylated allergens rVes v 1 and rVes v 5 as well as glycosylated allergens rVes v 2 and rVes v 3.

### Controls

Seventeen control subjects having been stung without adverse reactions were included in this study (Table 1).

### Intradermal skin test

All patients and controls were injected with 0.02 mL of yellow jacket venom 10–8, 10–6, 10–4 and 10–3 g/L i.c. on the volar side of the forearm as described previously [25]. A wheal of ≥5 mm in diameter with erythema at a concentration of ≤10–3 g/L was considered as a positive reaction.

### Generation of recombinant allergens

Genes of the recombinant allergens were cloned into modified Invitrogen expression vector. The vectors were transformed into electrochemical cells and transfected in High Five insect cells. Allergens were purified by Ni2+-affinity chromatography as previously described [26].

### Determination of specific serum IgE

Specific IgE to natural venom and the recombinant allergens Ves v 5 was determined in serum by ImmunoCAP *250* (Thermo Fisher, Uppsala). sIgE to recombinant allergens Ves v 1, Ves v 2 and Ves v 3 were analysed by BD ELISA kit (BD Biosciences, Heidelberg, Germany) as previously described [26].

### Basophil activation test

The basophil activation test was performed as described previously with modifications as recommended by the manufacturer of the assay (Flow-CAST; Bühlmann Laboratories, Basel, Switzerland). Basophils activation was evaluated by measuring the CD63 expression by flow cytometry (FACS calibur BD Biosciences) (15)

### Statistical Analysis

To define the cut-off values for clinical decision-making, ROC analysis were performed (Prism 5; Graph-pad software, La Jolla, Calif). Sensitivity and specificity were calculated as follow: Sensitivity  =  true-positive/(true-positive results + false-negative results) Specificity  =  true-negative/(true-negative results + false-positive results). Spearman test was used to evaluate correlations. Statistically significant differences were defined as *P<0.05, **P<0.01 and ***P<0.001.
